# Spatial Integration and the Underlying Mechanisms of Cross-Modality Interference

**DOI:** 10.5334/joc.5

**Published:** 2018-01-10

**Authors:** Danielle A. Lutfi-Proctor, Emily M. Elliott, Edward J. Golob

**Affiliations:** 1 Louisiana State University, US; 2 University of Texas at San Antonio, US

**Keywords:** Stroop effect, cross-modal Stroop, spatial attention, goal maintenance, cross-modal distraction, auditory distraction

## Abstract

Researchers have often utilized the classic Stroop task as a measure of selective attention processes. While it is largely agreed upon that semantic interference plays a role in the classic task, the role of attentional processes is less clear. The picture is further muddied when variations on the classic task are used. For example, the cross-modal Stroop task, in which one names the color of visual items while ignoring distracting auditory color words, typically leads to smaller sized interference effects and little or no facilitation when compared to the classic task. Furthermore, relationship(s) with working memory capacity have only been found in the classic version. We examined whether these differences are due to a methodological factor; namely, spatial integration versus separation of the target and distractor locations. We conducted four experiments manipulating the location of auditory distractors within the cross-modal Stroop task, and found that the location of the distractors had little to no impact on the size of the effect. This lack of an effect of location implies that the mechanisms contributing to the cross-modal Stroop effect are not the same as those of the classic Stroop effect, and that the role of spatial attention in cross-modal Stroop is limited. The implications of a unique role for auditory distractors is considered as well, and supports the conclusion that interference in the cross-modal Stroop effect is the result of a combination of semantic interference and modality-specific interference.

## Spatial Integration and the Underlying Mechanisms of Cross-Modality Interference

While sounds can, at times, help performance, other times the inability to completely avoid distraction can be a hindrance. Researchers have studied the flexibility of cognitive systems and found that many factors determine the likelihood of whether an irrelevant stimulus harms or helps performance. One consistent and well-studied example of a distracting stimulus harming performance is the classic Stroop task (Stroop, 1935).

The classic Stroop task involves naming the color of the ink of incongruent color words (e.g., the word *red* in blue font) or a nonverbal control stimulus such as a row of #’s (MacLeod, 1991; Stroop, 1935). It is typically found that incongruent trials display slower response times and more errors than control trials. Some researchers have described the cause of Stroop interference to be due to a combination of semantic and lexical interference arising from well-practiced reading behavior (e.g. Ferrand & Augustinova, 2014), while others have questioned the “automaticity” view of Stroop interference and instead have suggested that the interference is the result of a combination of word recognition and attentional processes, namely the allocation of spatial attention (e.g., Besner & Stolz, 1999). The current research examined the role of spatial attention in a cross-modal variant of the Stroop task to determine if the cross-modal version can be explained by similar underlying mechanisms as the classic Stroop effect, or if a different explanation is needed that draws upon characteristics of the auditory modality itself.

## Cross-Modal Stroop

Since the initial discovery of the classic Stroop effect, many different versions of the task have been created, including a picture-word version (Glaser & Düngelhoff, 1984; Meyer & Schriefers, 1991; Starreveld & La Heij, 2017), an auditory version (Green & Barbara, 1981; Green & Barbara, 1983), and a cross-modal version (Cowan & Barron, 1987; Elliott, Cowan, & Valle-Inclan, 1998). The cross-modal Stroop task involves naming the color of a visual item, typically a color square, while ignoring incongruent and congruent auditory color words. The control is usually a silent condition. Participants are unable to avoid the auditory distractors, even when instructed to ignore them, and this interference has been interpreted as a combination of factors, such as response competition and semantic interference (Cowan & Barron, 1987; Elliott et al., 1998). However, despite the fact that the effect is generally referred to as a “Stroop effect,” the results vary from those recorded in the classic version. Interference is found in the cross-modal version, but it is significantly smaller than that displayed in the classic version (approximately 90 ms vs. 240 ms; Elliott et al., 2014; note that these numbers are from experiments in which 75% of the trials were congruent. This manipulation increases the size of both the classic and cross-modal Stroop effects, however, the same general pattern appears to remain even when this manipulation is not used and the effects are smaller, such as a 60 ms cross-modal Stroop effect when equal portions of silence, non-color word, and color word trials were studied; Elliott et al., 1998).

Also, facilitation is rarely seen in the cross-modal version (Elliott et al., 2014; Francis, MacLeod, & Taylor, 2017; Lutfi-Proctor, Elliott, & Cowan, 2014; Morey et al., 2012; see Roelofs, 2005; Shimada, 1990, for exceptions with stimulus onset manipulations) but is relatively consistent in the classic version (MacLeod, 1991). Lastly, the classic version has been shown to have a relationship with working memory capacity (WMC; Hutchison, 2011; Kane & Engle, 2003; Long & Pratt, 2002), while no such relationship has been found with the size of the cross-modal Stroop effect (Elliott, Barrilleaux, & Cowan, 2006; Morey et al., 2012). Some researchers have suggested these differences, especially those related to WMC, are due to the relative importance of goal maintenance in each version of the task (Morey et al., 2012).

However, a methodological hypothesis is that the differences between the two effects are due to factors related to the presentation of the stimuli, as opposed to the modality per se (Francis et al., 2017). In classic Stroop, the target and distractor (the color and the written word) are typically presented in the same location in space. In the cross-modal Stroop task, the target is presented on a screen in front of the participant, while the distractor usually comes from a set of headphones or speakers. We explored this hypothesis regarding the spatial integration or separation of the target and distractor to determine whether this spatial component has an influence on the mechanisms of interference.

## Spatial Location and Classic Stroop

The integration of the written word and the color of the font has been shown to have an impact on the size of the classic Stroop effect. As one example, researchers have separated the written word and the color by superimposing a black word on a colored rectangle. These types of manipulations have produced relatively consistent, but significantly lower, levels of interference and facilitation. Moreover, the farther the written word and color square are presented from one another (e.g. the word is presented, 1, 5, or 10 cm above or below the color patch) the smaller the size of the effect (Flowers & Stoup, 1977; Kahneman & Chajczyk, 1983; Risko, Sotlz, & Besner, 2005).

We were interested in determining the importance of spatial integration versus separation of the targets and distractors in the cross-modal Stroop effect to understand the degree to which an attention-based mechanism is involved. Drawing upon past work in classic Stroop, there is evidence that some form of attention needs to be directed at the written word in order for it to have an impact. These include the importance of spatial integration, dilution (the fact that a color word has less of an impact on naming color stimuli if other word-like objects are displayed simultaneously) and the manipulation of coloring a single letter of the written color word. In other words, the less attention directed at the word, the less of an impact it has, thus decreasing the size of the Stroop interference effect. Spatial attention and attention capture are frequently considered candidate processes for this role (Chajut, Schupak, & Algom, 2009; Stolz & Besner, 1996; Besner & Stolz, 1999; Kahneman & Chajczyk, 1983; Labuschagne & Besner, 2015; Mitterer, La Heij, & Van der Hijden, 2003; Stolz & Besner, 1999; see Ferrand & Augustinova, 2014; Marmurek, 2003 for an opposing view). Moreover, the finding that a distractor has a larger impact if it is closer to a target is not exclusive to the classic Stroop task. Similar findings have been found in other visual paradigms, such as flanker tasks (Eriksen & Eriksen, 1974; Eriksen & Hoffman, 1972).

## Spatial Location of Visual Targets and Auditory Distractors

The importance of the location of a distractor does not appear to be limited to visual distraction paradigms. Spence et al. (2000) found that the ease with which a distracting sound can be ignored depends on the distance between fixation and attended visual effects. In a series of three experiments, Spence and colleagues had participants shadow an auditory stream coming from behind while fixating on a visual stream (lip movements or a series of meaningless shapes). At the same time, participants either passively or actively ignored an irrelevant stream of words from either the same or opposite side as fixation. They found a decrement in shadowing when participants passively fixated towards the irrelevant auditory stream. Furthermore, this decrement was even larger when participants performed a difficult active visual task verses simple fixation (Spence et al., 2000).

Similar results have also been found within the irrelevant-sound effect (ISE) literature. The ISE involves the impairment of immediate serial recall of visually presented lists when irrelevant auditory stimuli are presented during either encoding or retention (Buchner et al., 2008). Buchner et al. (2008) had participants memorize lists of visually presented digits in silence or while ignoring distractor sounds, which either came from in front of the participant and, therefore, from the direction in which the participants’ attention was oriented, or from behind. Buchner et al. (2008) found that while the distractor sounds impaired recall performance on the whole, the largest impairment was observed when the sound source was directionally close to the frontal visual target display (or to-the be remembered numbers). The authors interpreted this finding as an implication of the importance of attentional processes in determining the size of the distracting effect of auditory stimuli on the recall of visual stimuli.

Thus, evidence suggests that location can have an impact on how “distracting” a distractor is in a cross-modal scenario as well as a visual-only one. It is, therefore, possible that some of the differences between the cross-modal and classic Stroop effects could be due to previously inherent differences in experimental design.

## The Current Study

As mentioned above, the typical classic Stroop paradigm displays the target (the color) and distractor (written word) in the same location in space. The cross-modal Stroop task, meanwhile, generally displays the target on a computer screen in front of the participant and the distractors (the auditory color words) come from the location of the participant’s head through a set of headphones. As the location of the distractors has been shown to have an impact on distraction effects both in classic Stroop and in other cross-modal paradigms, it may be that at least some of the differences between the cross-modal and classic Stroop effects are due to the lack of spatial integration in cross-modal Stroop. This, in turn could lead to the sounds being less likely to capture attention and/or less spatial attention directed towards them, which would in turn reduce the size of the interference and facilitation effects from the auditory distractors.

We conducted a series of four experiments examining the role of spatial location in the cross-modal Stroop task. In Experiments 1 and 2, participants heard the auditory distractors either from headphones or speakers located next to the computer. Experiment 3 expanded the location manipulation and presented the sounds through speakers located either in front of the participant, next to them, or behind them, while Experiment 4 utilized spatialized sounds which were manipulated so that they sounded like they came from in front of the participant or from either the right or left side (Golob & Holmes, 2011). If spatial location determines the likelihood of a distractor to be attended to (at least in part) and increases the amount of distraction, one would expect the sounds which appear to be coming from a location closer to the target to have more of an impact and to increase the size of the cross-modal Stroop effect, relative to those sounds which are farther away. If the spatial location does not influence the size of the cross-modal Stroop effect, it would imply that spatial attention and/or attention capture are not playing the same role in the cross-modal Stroop effect as in the classic version. Furthermore, this pattern of findings would provide additional support for the view that differences between the classic and cross-modal versions of the task are driven, at least in part, by differences in goal maintenance requirements between the two versions of the task, as opposed to low-level differences in stimuli presentation.

## Experiments 1 and 2

These experiments were conducted to determine whether there was a difference in performance when the distracting sounds were presented from a location farther from the target (i.e. headphones) or from a location closer to the target (i.e. speakers in front of the participant next to the computer screen). If the location of the sound has no influence on the size of the effect, it would imply that the role of spatial attention and/or attention capture is not the same in the cross-modal Stroop effect as the classic Stroop effect.

Additionally, the issue of the proper control or neutral condition is relevant as well. Some cross-modal literature used tones as a control rather than silence (Elliott & Cowan, 2001; Francis et al., 2017; Shimada, 1990). If any sound can lead to a certain amount of distraction and interference, it is possible the cross-modal Stroop effect arises due to a combination of two types of interference: basic sound interference which leads to a base level of distraction regardless of its composition, and semantic interference, which can be either positive or negative based on its congruency. This is similar in concept to the theory that Stroop interference is due to a combination of semantic and lexical interference arising from well-practiced reading behavior (e.g. Ferrand & Augustinova, 2014). If the cross-modal Stroop effect is caused by dual mechanisms, one would expect the introduction of a tone as the control to lessen the size of the cross-modal Stroop; the difference between the control and incongruent and congruent trials would largely relate to the semantic mechanism. In addition, if attentional mechanisms do play a role in the cross-modal Stroop effect, one would still expect to see an impact of spatial location on the size of the cross-modal Stroop effect.

## Method

### Participants

Participants in Experiment 1 consisted of 67 undergraduates (66% female; *M* = 20.89 years, *SD* = 3.10) who participated for course or extra credit. Participants who were hearing impaired, did not have English as a first language, or were color blind were not eligible to participate. Four students were dropped from all data analyses (final N = 63).

Participants in Experiment 2 were 72 Louisiana State University undergraduates. Three students were dropped from all data analyses due to not meeting the exclusionary requirements (final N = 68; 88%, *M* = 19.84 years, *SD* = 3.08).

### Materials and Design

These experiments consisted of two cross-modal (CM) Stroop tasks (within-subjects): Speaker CM Stroop and Headphones CM Stroop. In Speaker CM Stroop, the auditory distractor was presented from a pair of speakers located next to the computer screen, while in Headphones CM Stroop, the auditory distractors were heard from a set of over-the-ear headphones. The order of the two tasks (between-subjects) was counterbalanced to control for any potential impact of task order on performance. Congruent, incongruent, and control trials were presented to examine both interference and facilitation (within-subjects).

Each task was completed in a separate block and began with nine practice trials followed by 162 experimental trials. The proportion of congruent, incongruent, and control trials was equal in both tasks – each auditory distraction condition was presented 54 times – and randomly intermixed.

Squares were used as the to-be-named color carrying stimulus, and the auditory color words were a recorded female voice presented at a subjectively equal volume. There were three colors used throughout the entire experiment: red, blue, and green. These colors created six incongruent color combinations (*red-*green, *red-*blue, *blue-*red, *blue-*green, *green-*red, and *green-*blue) – each used nine times in each task – as well as three possible congruent pairings (*red-*red, *blue-*blue, and *green-*green) – seen 18 times in each task.

Each trial began with a fixation cross in the center of the screen which remained for 500 ms followed by a color square. The onset of the sounds and color squares were simultaneous (there was no auditory distractor for control trials in Experiment 1, and in Experiment 2 it was a 500 ms tone). Participants were instructed to name the color of the square as quickly and accurately as possible and were asked not to wait for the auditory word to finish before initiating the response. A microphone connected to a response box logged the vocal onsets and response times of the participants. After the participant named the color, the experimenter answered three questions: what response was given by the participant, whether a false start had taken place (the microphone was triggered before the participant was able to give their response), and whether they had made an error in answering the first two questions.

The experiments were presented using E-Prime 2.10 software on a Dell Dimension desktop computer with a 17-inch monitor. Participants were tested one at a time, and the experiments took approximately 20 minutes to complete.

## Results

A total of 0.48% trials were removed due to response errors (see Table A1 in the Appendix), 3.53% because of false starts, and 0.31% for experimenter errors in Experiment 1. For all of the analyses in all of the experiments α = 0.05, and the Bonferroni correction was used for follow-up tests. In cases where sphericity was violated and the results were significant, the Greenhouse-Geisser correction was used.

In Experiment 2, the overall error rates were also low. A total of 1.38% trials were removed due to response errors (see Table A1 in the Appendix), 1.96% because of false starts, and 0.08% for experimenter errors.

### Response Times for Experiment 1

**Figure 1:**
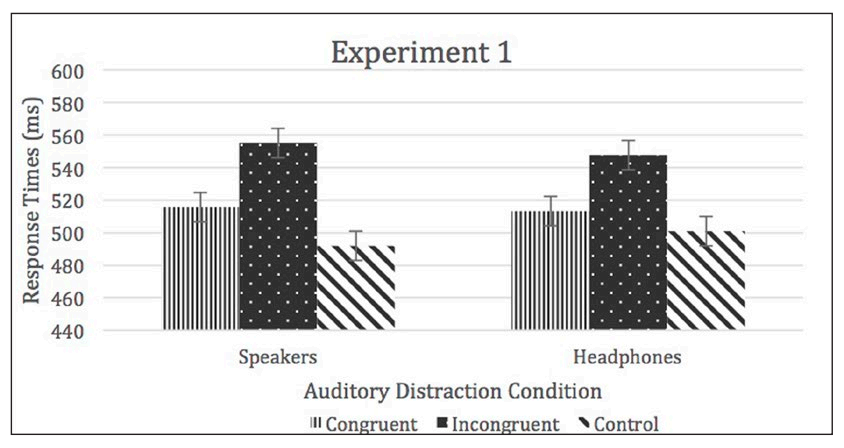
RTs for Experiment 1. Error bars represent within-subject confidence intervals.

A 2 (sound location) × 3 (auditory distraction) repeated-measures ANOVA was used to analyze the means of medians of RTs.^1^ There was no main effect of sound location, η^2^_p_ < 0.01, but there was a main effect of auditory distraction condition, *F* (2, 124) = 103.56, *p* < 0.05, η^2^_p_ = 0.63 (incongruent > congruent > control), which was qualified by a location by auditory distraction interaction, *F* (2, 124) = 9.73, *p* < 0.05, η^2^_p_ = 0.14 (see Figure 1).

**Figure 2:**
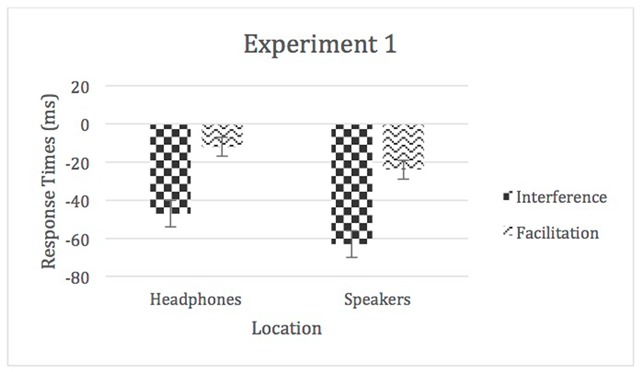
Facilitation and interference scores for Experiment 1. Error bars represent within-subject confidence intervals.

In order to analyze this interaction, incongruent and congruent “interference” and “facilitation” scores were created. This was done by subtracting each participant’s incongruent and congruent median from his or her control median respectively. In this experiment, both incongruent and congruent trials were slower than control ones, thus, both display negative scores. Overall congruent trials would be expected to be positive and, thereby, display typical “facilitation” results, which would mean they were faster than control trials. The interference and facilitation scores were analyzed with a 2 (location) × 2 (auditory distraction) repeated-measures ANOVA. The amount of interference/facilitation significantly varied by location, *F* (1, 62) = 17.33, *p* < 0.05, η^2^_p_ = 0.22, with speakers > headphones. There was also a main effect of auditory distraction, *F* (1, 62) = 110.29, *p* < 0.05, η^2^_p_ = 0.64, with interference > facilitation; however, the auditory distraction by location interaction was not significant, η^2^_p_ = 0.03 (see Figure 2).

### Response Times for Experiment 2

**Figure 3:**
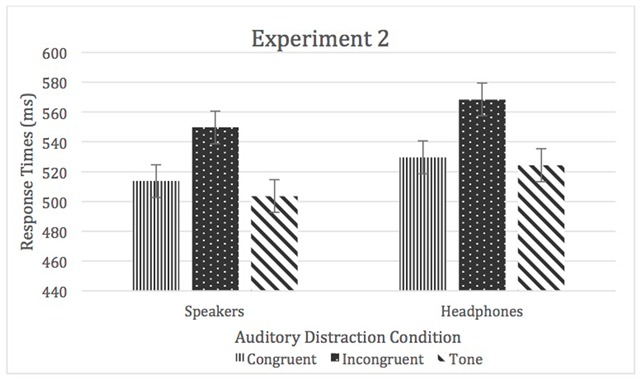
RTs for Experiment 2. Error bars represent within-subject confidence intervals.

A 2 (task order) × 2 (location) × 3 (auditory distraction) mixed-modal ANOVA was used to analyze the means of medians of RTs. There was a main effect of sound location, *F* (1, 66) = 5.34, *p* < 0.05, η^2^_p_ = 0.08 (headphones > speakers), as well as auditory distraction, *F* (2, 132) = 72.59, *p* < 0.05, η^2^_p_ = 0.52 (incongruent > congruent = control), and a significant main effect of task order, *F* (1, 66) = 6.19, *p* < 0.05, η^2^_p_ = 0.09 (speakers first > headphones first). None of the interactions were significant. In all cases η^2^_p_ < 0.01 (see Figure 3).

**Figure 4:**
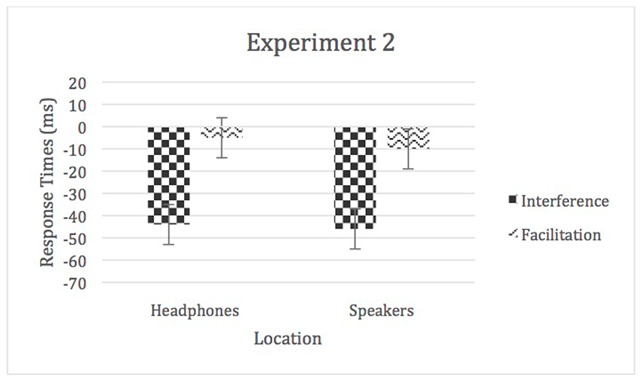
Facilitation and interference scores for Experiment 2. Error bars represent within-subject confidence intervals.

As in Experiment 1, we removed task order for further analysis of the RT data, and also created “interference” and “facilitation” scores for congruent and incongruent trials in both the speakers and headphones condition and analyzed them with a 2 (location) × 2 (auditory distraction) repeated-measures ANOVA. There was a significant main effect of auditory distraction, *F* (1, 67) = 93.12, *p* < 0.05, η^2^_p_ = 0.58 (interference > facilitation); however, there was no main effect of location or a significant location by auditory distraction interaction (in both cases η^2^_p_ < 0.01; see Figure 4).

## Discussion

The results from Experiment 1 suggested that spatial location may have an impact on the size of the cross-modal Stroop effect. Participants were slower to respond when the sound was coming from speakers next to the computer and, therefore, closer to the target, than when the sound was coming from headphones. However, we also found that congruent trials were significantly slower than control trials in terms of their RTs and this slowing was even greater in the speaker condition than the headphones one.

In contrast to Experiment 1, the results from Experiment 2 indicated that the spatial location of the targets and distractors did not have any impact on the size of the cross-modal Stroop effect. Moreover, the headphones condition produced slower RTs overall than the speaker condition.

It is possible that the use of a tone decreased the size of the cross-modal Stroop effect to the point that the impact of spatial location became difficult to determine. However, given that both the headphone and speaker conditions in Experiment 2 produced a mean level of interference (44 ms and 46 ms respectively) equivalent to that of the headphone level in Experiment 1 (47 ms), this seems unlikely. Given the inconsistent findings across Experiments 1 and 2, we conducted a third experiment to examine the role of spatial location on the size of the cross-modal Stroop effect.

Research examining audition in general has suggested that the spread of auditory attention is broader in scope than that of visual attention (Golob & Holmes, 2011; Mock et al. 2015). Humans generally have a 180° angle of vision and, as such, not all of the visual environment surrounding a person can processed at once. Due to this, visual attention does not extend past what the eyes can perceive, or, roughly, 180°. We are, however, capable of hearing sounds from all around us. Therefore, a sound coming from behind us can capture our attention even if a blinking light behind us would not. It is possible that our spatial location manipulation was not strong or broad enough to find consistent results. The difference between a central square and a word located on the edge of the screen may not be the equivalent of a color square on a screen and auditory words coming from headphones, but instead be the comparable to a color square located on a screen and auditory words coming from behind a participant.

To address these concerns, three spatial locations were used and the control condition was returned to silence. The sound was presented from speakers next to the computer (this was identical to the speakers condition in Experiment 1 and 2), from speakers located on either side of the participant, and from speakers behind the participant.

## Experiment 3

Previous research involving spatial location and auditory distraction has used sounds coming from behind the participant (Buchner et al., 2008) or from the opposite side of the room (Spence et al., 2000). In this experiment, we increased the spatial location manipulation to determine if broadening the scope of spatial location had any impact on the size of the cross-modal Stoop effect.

## Method

### Participants

Ninety-four Louisiana State University undergraduates (86% female; *M* = 19.90 years, SD = 1.60) participated for course or extra credit. Four students were dropped from all data analyses for not meeting the requirements and one due to a data collection error (final N = 89).

### Materials and Design

The stimuli and procedure were nearly identical to those used in Experiment 1, except the location of the auditory distractor was altered to consist of three levels instead of two. In this experiment, the sound came from speakers behind the participant (behind), from speakers set-up on either side of the participant (besides), or from speakers next to the computer (front). Each sound location was presented in its own block, and the order of the blocks was controlled for by a Latin square. Each block consisted of nine practice trials followed by 162 experimental trials. The proportion of congruent, incongruent, and control trials was equal in all blocks – each auditory distraction condition was presented 54 times – and randomly intermixed. There was a total of 486 experimental trials. The task took approximately 30 minutes to complete.

## Results

Consistent with the first two experiments, error rates were low. A total of 0.70% trials were removed due to response errors (see Table A2 in the Appendix), 1.02% because of false starts, and 0.17% for experimenter errors.

### Response Times

**Figure 5:**
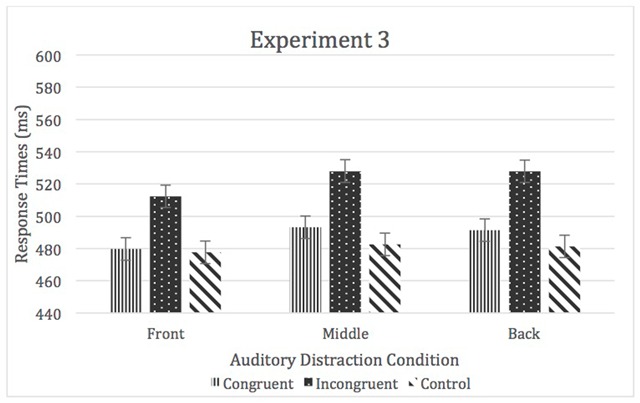
RTs for Experiment 3. Error bars represent within-subject confidence intervals.

A 3 (task order) × 3 (location) × 3 (auditory distraction) mixed-model ANOVA was used to analyze the means of medians. There was a significant main effect of location, *F* (2, 170) = 3.74, *p* < .05, η^2^_p_ = 0.04, however none of the follow-up pairwise comparisons were significant. There was also a significant main effect of auditory distraction, *F* (1.75, 148.33) = 113.23, *p* < .05, η^2^_p_ = 0.57, incongruent > congruent = neutral. Moreover, while the location by auditory distraction interaction, η^2^_p_ = 0.03, and main effect of task order, η^2^_p_ < 0.01, were not significant, the three-way interaction of location, auditory distraction, and task order was significant, *F* (8, 340) = 2.97, *p* < .05, η^2^_p_ = 0.07 (see Figure 5).

In order to examine the significant three-way interaction, interference and facilitation scores were created for each location for each task version. For Version A (Front, Middle, Back) location had a significant impact on the amount of interference, *F* (1.53, 44.22) = 6.39, *p* < .05, η^2^_p_ = 0.18, with front < middle = back. There was also a significant main effect of location for facilitation, *F* (2, 58) = 5.03, *p* = .05, η^2^_p_ = 0.15, with front < back with middle not different from either.

For Version B (Middle, Back, Front) there was no significant effect of location on interference, η^2^_p_ = 0.09; however, location did have a significant impact on facilitation, *F* (2, 54) = 3.35, *p* < .05, η^2^_p_ = 0.11, with middle < back with front not being significantly different from either. Lastly, for Version C (Back, Front, Middle) there was no significant main effect of location for interference, η^2^_p_ = 0.04, nor a significant impact of location on facilitation, η^2^_p_ = 0.07 (see Table 1).

**Table 1:** Interference and Facilitation Scores for Experiment 3. Standard deviations are displayed in the parentheses.

Version	Location	Interference	Facilitation

	Front	14.5 (65.34)	10.58 (49.69)
Version A	Middle	42.92 (26.85)	–10.60 (30.24)
	Back	44.80 (33.84)	–8.53 (32.84)
	Front	48.48 (35.61)	–13.98 (37.37)
Version B	Middle	41.04 (33.23)	–7.96(29.29)
	Back	55.88 (38.27)	–22.52 (30.37)
	Front	42.37 (39.90)	–3.52 (37.62)
Version C	Middle	51.98 (37.83)	–12.98 (44.68)
	Back	39.67 (33.97)	0.02 (37.84)

## Discussion

Three spatial locations were utilized in Experiment 3 to increase the range of locations of the distracting auditory stimuli. However, while significant results were observed, the direction of the findings was not consistent with the findings of Experiment 1. In addition to the lack of consistency across Experiments 1–3 in regards to the effects of location order, within Experiment 3 the effect of location order was inconsistent across RT and error data. Given these inconsistencies, in order to try to clarify the impact of location on the size of the cross-modal Stroop effect, we ran an additional study utilizing spatialized sounds (Golob & Holmes, 2011) and returned to tones as the control condition. Spatialized sounds are auditory stimuli that are modified to simulate the natural cues to localize a sound in space (Blauert, 1997), and are delivered through insert headphones. The result is a sound that appears to be coming from a particular 3-D location even though the sounds are really coming from the headphones. Therefore, participants can hear a sound that appears to be coming from in front of or next to them without having to use speakers, and without the concern that the participant could be moving their head and changing the relative location of the sounds, due to head position.

## Experiment 4

Unlike sounds from speakers, the virtual location of sounds limits the influence of visual indicators of sound sources. By utilizing sounds that were manipulated so that they appeared to be coming from a particular location, we were able to explore the impact of spatial location when visual markers were not available and to examine whether these spatialized sounds had an impact when speakers located at various locations did not. The use of a tone for a control condition was reintroduced to determine if the location of the “control” tone had any impact on the overall results.

## Method

### Participants

Sixty-six Louisiana State University undergraduates participated for course or extra credit. Thirteen students were dropped from all data analyses for not meeting these requirements (final N = 53; 81% female; *M* = 20.72 years, *SD* = 4.50).

### Materials and Design

**Figure 6:**
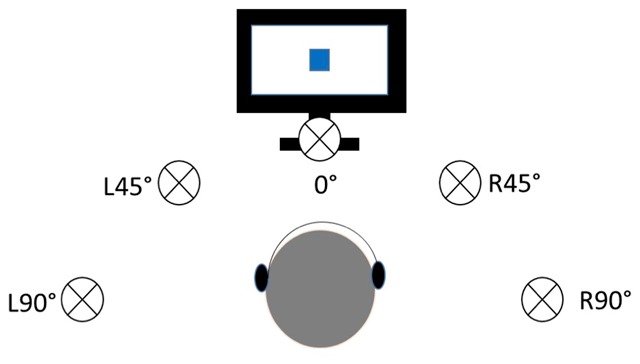
Visual depiction of the location of the spatialized sounds.

In this experiment, the sounds were presented in a male voice and were spatialized so that they appeared to come from a 90° or 45° angle to the left (L90° and L45° respectively), a 90° or 45° angle to the right (R90° and R45° respectively), or from directly in front of the participant (0°; see Figure 6). Spatialized sounds were created by applying interaural time and level differences as well as head-related transfer functions that corresponded to those of the five locations (Tucker Davis, Gainesville, FL). Previous work shows that perception of spatialized binaural stimuli is comparable to the perception of sound sources in free field (Wightman & Kistler, 1989). The sounds were presented to the participant through a set of Etymotic ER-3C insert earphones. The location of the sounds was blocked (e.g., participants experienced auditory distractors coming from only a +90° angle, then from 0°, etc.). The order of the blocks was randomly selected to control for order effects, and participants were informed at the start of each block whether the sounds would be coming from the right, left, or in front of them.

The experiment consisted of a 5 (location: L90°, L45°, 0°, R45°, R90°) × 3 (auditory distraction) within-subjects design. The proportion of congruent, to incongruent, to control trials was equal – each auditory distraction condition was presented 120 times over the course of the experiment. Each location was presented 72 times, and each auditory distraction condition was presented at each location 24 times. There were a total of 360 experimental trials and 45 practice trials (there were 9 practice trials before each block). The task took approximately 30 minutes to complete.

## Results

Error rates were low. A total of 0.68% trials were removed due to response errors (see Table A3 in the Appendix), 1.71% because of false starts, and 0.21% for experimenter errors.

### Response Times

**Figure 7:**
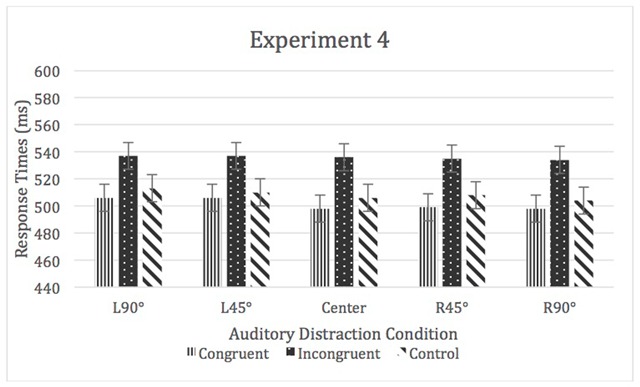
RTs for Experiment 4. Error bars represent within-subject confidence intervals.

**Figure 8:**
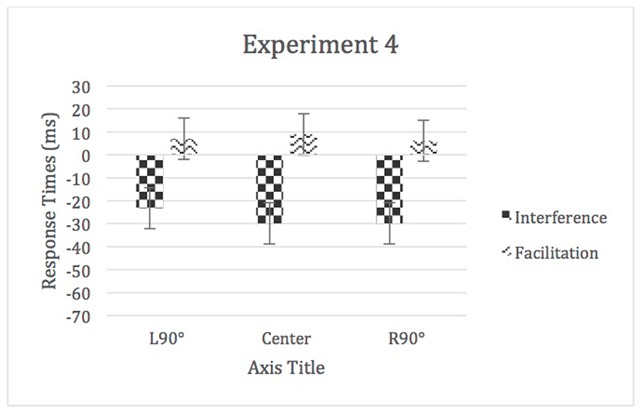
RTs for Experiment 4. Error bars represent within-subject confidence intervals.

A 3 (auditory distraction) × 5 (location) repeated-measures ANOVA was used to analyze the means of medians. There was a significant main effect of auditory distraction, *F* (1.73, 89.68) = 77.99, *p* < .05, η^2^_p_ = 0.60. There was no main effect of location, η^2^_p_ = 0.01, nor a significant location by auditory distraction interaction, η^2^_p_ = 0.01 (see Figures 7 and 8).

## Discussion

The results from Experiment 4 provide more evidence that acoustic spatial location has little, if any, impact on the size of the cross-modal Stroop effect. One possible concern with the use of spatialized sounds to create the perception of individual locations was that participants did seem to have some difficulty determining the sound locations. Every participant was asked to fill out a sheet at the end of the experiment in which they listened to the stimuli used in the experiment and reported where they thought the sound was coming from. However, even when the participants who did exceptionally badly on this were removed (i.e. they placed all of the sounds on the left side of space when at least some should have been on the right), the results remained the same.

Spatial hearing is normally variable even when delivered from speakers in free field (Lewald, Dörrscheidt, & Ehrenstein, 2000), and often improves with brief periods of practice (Mendonca et al., 2012). We also conducted two additional studies utilizing the same spatialized sounds. One of the studies was identical to the one described here except that over the ear headphones were used to present the spatialized sounds. The other study also used over the ear headphones and the location conditions were randomly intermixed rather than blocked. The results from both of these studies are virtually identical to the ones listed here. Location failed to have any impact on the size of the cross-modal Stroop effect. Nonetheless, it is possible that the removal of the “bad” participants dropped power to a level that these minute location variations were no longer apparent.

Given the nature of null hypothesis testing, one cannot say that a non-significant finding means that there is no difference between the groups. We also conducted Bayesian analyses on all four experiments in an attempt to help clarify the impact of location on the size of the cross-modal Stroop effect.

## Bayesian Analyses

**Figure 9:**
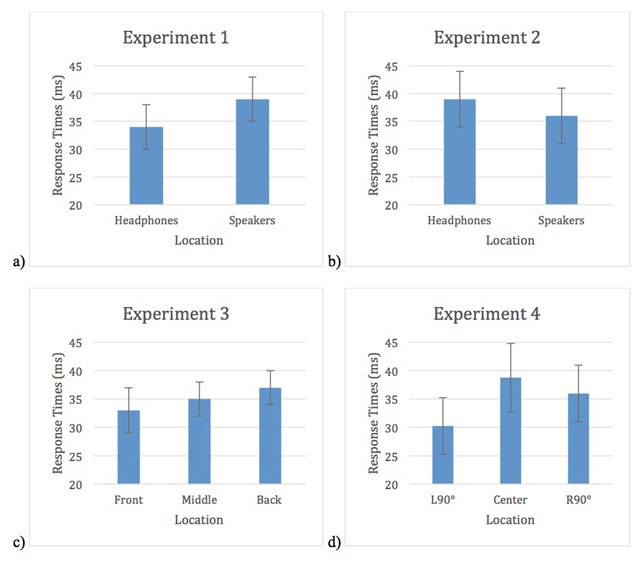
Difference scores for **a)** Experiment 1, **b)** Experiment 2, **c)** Experiment 3, and **d)** Experiment 4. Error bars represent standard errors.

The JZS Bayes factor compares the degree to which the data support the alternative hypothesis (the standardized differences in the means is not zero and is distributed as a *t* with 1 degree of freedom) versus the null hypothesis (there is no difference in means). We conducted paired samples *t*-tests to compare the difference scores (incongruent mean – congruent mean) for the different locations for each experiment. Difference scores were used because the control condition differed across all four experiments (in Experiment 1 and 3 the control condition consisted of silence while in Experiments 2 and 4 a tone was used). We then used the t-value as the basis for the odds derivation using the calculator available on: http://pcl.missouri.edu/bayesfactor. The full results are reported in Appendix B and the difference scores are depicted in Figure 9.

Altogether, the Bayesian analyses provide support for the null hypothesis and the idea that the location of the auditory distractors is not having an impact on the size of the cross-modal Stroop effect. This is not consistent with the findings from the classic Stroop task, and provides evidence that the same mechanisms are not involved in both versions of the task. This is not to say that there is no overlap whatsoever in the mechanisms for both versions, but that one cannot assume that they are due exclusively to the same underlying causes.

## General Discussion

We were interested in whether the differences between performance in the cross-modal and classic Stroop paradigms (e.g., the amount of interference and facilitation induced by distractors, and a relationship or lack thereof with working memory capacity) are due, at least in part, to a basic methodological difference in the ways the two tasks are conducted (see Francis et al., 2017). In classic Stroop the target and distractor are generally presented in the same location, while cross-modal Stroop typically presents the target on a screen in front of the participant while the distractor is heard through a set of headphones. Research both on the Stroop task (Flowers & Stoup, 1977; Kahneman & Chajczyk, 1983; Risko et al., 2005) and other audio-visual cross-modal paradigms (Buchner et al., 2008; Francis et al., 2017; Spence et al., 2000) have suggested that the spatial integration of a target and distractor increases the likelihood that the distractor will have an impact on performance. This finding is usually interpreted to mean that spatial attention and/or attention capture is playing a role in an effect.

In order to examine whether the differences between the findings of cross-modal and classic Stroop are due to the impact of spatial integration, or spatial attention, we conducted a series of four experiments in which we manipulated the location of the auditory distractors in a cross-modal Stroop task. We also varied the control condition and used silence in Experiments 1 and 3, and tones in Experiments 2 and 4.

Across the set of four experiments, only one of them, Experiment 1, showed any evidence that spatial location had an impact on the size of the cross-modal Stroop effect. Moreover, when we re-ran the analyses using Bayesian statistics, none of the experiments, including Experiment 1, showed an impact of spatial location. Furthermore, it does not appear that our studies were simply underpowered. Before conducting the experiments, power analyses were performed. As all of our experiments were repeated measure designs, and, following prior work indicating that the effect of location was “medium” in nature (Buchner et al., 2008), we had more participants than necessary for a probability of 1 – β = .95. In fact, assuming we had been dealing with a medium effect size (*f* = .25; Cohen, 1977) of location as expected, with our actual participant numbers, an assumed correlation between two levels of the repeated-measures variable of ρ = .5, and an α = .05, all of our experiments had a probability of 1 – β = .98 or higher depending on the experiment.

Overall, this suggests that spatial location is having a very small, to zero, impact on the size of the cross-modal Stroop effect, which is in conflict to what is found in the classic version of the task (Flowers & Stoup, 1977; Kahneman & Chajczyk, 1983; MacLeod, 1991; Risko et al., 2005). This finding suggests that at least some of the underlying mechanisms involved in cross-modal Stroop are not the same as those in classic Stroop, and that differences in goal maintenance, combined with aspects of modality-specific interference from the auditory channel, is the primary hypothesis that has support at this time. In classic Stroop the color and word are integrated; thus, when one looks at the color, it is very easy to forget the task-goal and accidently read the word instead. In contrast, in cross-modal Stroop one looks at a color square and there is nothing else one can do aside from name the shape as the auditory word is presented and then disappears. Therefore, in cross-modal Stroop it may simply be easier to maintain the task goal, leading to the results typically produced. This hypothesis needs to be investigated further to determine the relationships between the two types of Stroop tasks and the role of goal maintenance, to gain a better understanding of the potential implications for the executive processes engaged by the classic and cross-modal Stroop tasks.

Another explanation that has yet to be examined is that the process of generating the name of a color may not be impacted by the spatial location of auditory distractors in the same manner as other cross-modal tasks. Previous research examining audio-visual spatial location interference has involved either memory tasks (Buchner et al., 2008) or visual tasks (Spence et al., 2000). The overlap in the relevant processing of the distracting auditory stimuli with the processing of the target stimuli may differ in critical ways across these different methodologies (see Hughes, 2014); thus, potentially explaining the different findings regarding spatial location from those paradigms and cross-modal Stroop. As research examining the importance of spatial location on cross-modal distraction has been limited, we must continue to study cross-modal paradigms in order to determine whether they are affected in the same way.

However, it may be premature to say that spatial location has no impact whatsoever on the size of the cross-modal Stroop effect at this time. As mentioned above, there may be two underlying major mechanisms leading to the cross-modal Stroop effect: 1) a basic sound distraction mechanism or the fact that any sound, especially after hearing silence, captures attention and leads to a detriment in performance (such as the orienting response; Cowan, 1995; Sokolov, 1963), and 2) semantic interference (e.g., Ferrand & Augustinova, 2014). It is possible that only one of these mechanisms, for instance the orienting response, is impacted by location. Experiment 1 was the only experiment that had a significant effect of spatial location on the cross-modal Stroop effect, and was one of only two experiments in which we used a control of silence. If a participant at times hears nothing, the trials with sounds capture attention to a larger degree than when the sounds are constant. Prior research suggests that attentional orienting habituates, leading to lessened or no attention capture occurring and potentially no impact of spatial location (habituation of the orienting response; Cowan, 1995; Elliott & Cowan, 2001; Lutfi-Proctor et al., 2014; Sokolov, 1963). In the future, by increasing the magnitude of the Stroop effect, perhaps by a congruency proportion manipulation, and by using a control of silent versus tone as a direct manipulation, we can determine the evidence for two separate mechanisms and if they are independently affected.

An alternative explanation is the joint influence account (MacLeod & Bors, 2002; Francis et al., 2017), in which multiple distractors can be processed, as opposed to a simple attention capture account (e.g., Kahneman & Chajczyk, 1983). The empirical findings supporting the joint influence account suggest that the differences between the classic Stroop paradigm and the cross-modal Stroop paradigm are driven primarily by the spatial integration versus separation of the target and distractor, as opposed to a view espousing modality differences. However, to clearly determine the role of modality, additional experiments contrasting the visual-only Stroop task with the auditory-only Stroop task need to be conducted with manipulations of integrated versus separated stimuli.

In conclusion, the spatial location of the auditory distractors in cross-modal Stroop has little to no impact on the size of the effect. This suggests that the same underlying mechanisms may not be causing the cross-modal and classic Stroop effects. This does not mean none of the mechanisms overlap, but simply that one cannot assume that all of the same mechanisms are involved. In addition, more work is needed to understand the impact of using a control of silence and a control which includes a sound, as well as contrasting the auditory and visual modality versions of the Stroop task. Future research will ultimately help us to understand the mechanisms involved in cross-modal Stroop and how they compare with those of classic Stroop in the visual modality.

## Data Accessibility Statement

The data used in the present article are accessible at https://osf.io/g7pe3/.

## Additional Files

The Additional files for this article can be found as follows:

10.5334/joc.5.s1 Appendix A. Error Rate Analyses.

10.5334/joc.5.s2 Appendix B. Bayesian Analyses.

## Footnotes

A mixed-model ANOVA was conducted to determine whether task order had any effect on the RT results. As it did not, this factor was removed from the remaining RT analyses.

## References

[1] Besner D., Stolz J. A. (1999). What kind of attention modulates the Stroop effect?. Psychonomic Bulletin & Review.

[2] Blauert J. (1997). Spatial Hearing: The Psychophysics of Human Sound Localization.

[3] Buchner A., Bell R., Rothermund K., Wentura D. (2008). Sound source location modulates the irrelevant-sound effect. Memory & Cognition.

[4] Chajut E., Schupak A., Algom D. (2009). Are spatial and dimensional attention separate? Evidence from Posner, Stroop, and Eriksen tasks. Memory & Cognition.

[5] Cohen J. (1977). Statistical power analysis for the behavioral sciences.

[6] Cowan N. (1995). Attention and memory: An integrated framework.

[7] Cowan N., Barron A. (1987). Cross-modal, auditory-visual Stroop interference and possible implications for speech memory. Perception and Psychophysics.

[8] Elliott E. M., Barrileaux K. M., Cowan N. (2006). Individual differences in the ability to avoid distracting sounds. European Journal of Cognitive Psychology.

[9] Elliott E. M., Cowan N. (2001). Habituation to auditory distractors in a cross-modal, color–word interference task. Journal of Experimental Psychology: Learning, Memory, and Cognition.

[10] Elliott E. M., Cowan N., Valle-Inclan F. (1998). The nature of cross-modal, color-word interference effects. Perception and Psychophysics.

[11] Elliott E. M., Morey C. C., Morey R. D., Eaves S. D., Shelton J. T., Lutfi-Proctor D. A. (2014). The role of modality: Auditory and visual distractors in Stroop interference. Journal of Cognitive Psychology.

[12] Eriksen B. A., Eriksen C. W. (1974). Effects of noise letters upon the identification of a target letter in a nonsearch task. Perception & Psychophysics.

[13] Eriksen C. W., Hoffanan J. E. (1972). Temporal and spatial characteristics of selective encoding from visual displays. Perception & Psychophysics.

[14] Ferrand L., Augustinova M. (2014). Differential effects of viewing position on standard versus semantic Stroop interference. Psychonomic Bulletin and Review.

[15] Flowers J. H., Stoup C. M. (1977). Selective attention between words, shapes and colors in speeded classification and vocalization tasks. Memory and Cognition.

[16] Francis W. S., MacLeod C. M., Taylor R. S. (2017). Joint influence of visual and auditory words in the Stroop task. Attention, Perception, and Psychophysics.

[17] Glaser W. R., Düngelhoff F.-J. (1984). The time course of picture-word interference. Journal of Experimental Psychology: Human Perception and Performance.

[18] Golob E. J., Holmes J. L. (2011). Cortical mechanisms of auditory spatial attention in a target detection task. Brain Research.

[19] Green E. J., Barber P. J. (1983). Interference effects in an auditory Stroop task: Congruence and correspondence. Acta Psychologica.

[20] Hughes R. W. (2014). Auditory distraction: A duplex-mechanism account. PsyCh.

[21] Hutchison K. A. (2011). The interactive effects of listwide control, item-based control, and working memory capacity on Stroop performance. Journal of Experimental Psychology: Learning, Memory, and Cognition.

[22] Kahneman D., Chajczyk D. (1983). Tests of the automaticity of reading: Dilution of Stroop effects by color-irrelevant stimuli. Journal of Experimental Psychology: Human Perception and Performance.

[23] Kane M. J., Engle R. W. (2003). Working-memory capacity and the control of attention: The contributions of goal neglects, response competition, and task set to Stroop interference. Journal of Experimental Psychology: General.

[24] Labuschagne E. M., Besner D. (2015). Automaticity revisited: When print doesn’t activate semantics. Frontiers in Psychology.

[25] Lewald J., Dörrscheidt G. J., Ehrenstein W. H. (2000). Sound localization with eccentric head position. Behavioural Brain Research.

[26] Long D. L., Prat C. S. (2002). Working memory and Stroop interference: An individual differences investigation. Memory & Cognition.

[27] Lutfi-Proctor D. A., Elliott E. M., Cowan N. (2014). The role of visual stimuli in cross-modal Stroop interference. PsyCh Journal.

[28] MacLeod C. M. (1991). Half a century of research on the Stroop effect: An integrative review. Psychological Bulletin.

[29] MacLeod C. M., Bors D. A. (2002). Presenting two color words on a single Stroop trial: Evidence for join influence, not capture. Memory and Cognition.

[30] Marmurek H. H. C. (2003). Coloring only a single letter does not eliminate color-word interference in a vocal-response Stroop task: Automaticity revealed. The Journal of General Psychology.

[31] Mendonça C., Campos G., Dias P., Vieira J., Ferreira J. P., Santos J. A. (2012). On the improvement of localization accuracy with non-individualized HRTF-based sounds. AES: Journal of the Audio Engineering Society.

[32] Meyer A. S., Schriefers H. (1991). Phonological facilitation in picture-word interference experiments: Effects of stimulus onset asynchrony and types of interfering stimuli. Journal of Experimental Psychology: Learning, Memory, and Cognition.

[33] Mitterer H., La Heij W., Van der Heijden A. H. C. (2003). Stroop dilution but not word-processing dilution: Evidence for attention capture. Psychological Research.

[34] Mock J. R., Seay M. J., Charney D. R., Holmes J. L., Golob E. J. (2015). Rapid cortical dynamics associated with auditory spatial attention gradients. Frontiers in Neuroscience.

[35] Morey C. C., Elliott E. M., Wiggers J., Eaves S. D., Shelton J. T., Mall J. T. (2012). Goal-neglect links Stroop interference with working memory capacity. Acta Psychologica.

[36] Risko E. F., Stolz J. A., Besner D. (2005). Basic processes in reading: Is visual word recognition obligatory?. Psychonomic Bulletin & Review.

[37] Roelofs A. (2005). The visual-auditory color-word Stroop asymmetry and its time course. Memory & Cognition.

[38] Shimada H. (1990). Effect of auditory presentation of words on color naming: The intermodal Stroop effect. Perceptual and Motor Skills.

[39] Sokolov Y. N. (1963). Perception and the conditioned reflex.

[40] Spence C., Ranson J., Driver J. (2000). Cross-modal selective attention: On the difficulty of ignoring sounds at the locus of visual attention. Perception and Psychophysics.

[41] Starreveld P. A., La Heij W. (2017). Picture-word interference is a Stroop effect: A theoretical analysis and new empirical findings. Psychonomic Bulletin & Review.

[42] Stolz J. A., Besner D. (1996). The role of set in visual word recognition: Activation and activation blocking as nonautomatic processes. Journal of Experimental Psychology: Human Perception and Performance.

[43] Stolz J. A., Besner D. (1999). On the myth of automatic semantic activation in reading. Current Directions in Psychological Science.

[44] Stroop J. R. (1935). Studies of interference in serial verbal reactions. Journal of Experimental Psychology.

[45] Wightman F. L., Kistler D. J. (1989). Headphone simulation of free-field listening. II: Psychophysical validation. Journal of the Acoustical Society of America.

