# Supplementary material for: Spatial Integration and the Underlying Mechanisms of Cross-Modality Interference
Source: J Cogn. 2018 Jan 10;2(1):2. doi: 10.5334/joc.5 (PMC6646940; doi:10.5334/joc.5)
Supplement: Appendix A. — Error Rate Analyses. [file joc-1-1-5-s1.pdf]

Appendix A

*Error Rate Analyses*

**Experiment 1**

In contrast to the RT analyses, task order did have a significant impact on errors; therefore, the results of the 2 (task order) x 2 (location) x 3 (auditory distraction) mixed-model ANOVA are reported. While there were no significant main effects of task order, $\eta^2_p = 0.01$, or location, $\eta^2_p = 0.01$; there was a significant main effect of auditory distraction, $F$ (1.78, 108.69) = 6.69, $p < 0.01$, $\eta^2_p = 0.10$, with incongruent > congruent = control. Furthermore, while the auditory distraction by task order interaction, $\eta^2_p = 0.02$, location by auditory distraction interaction, $\eta^2_p < 0.01$, and auditory distraction, by task order, by location interactions $\eta^2_p = 0.03$, were not significant, the location by task order interaction was significant, $F$ (1, 61) = 6.77, $p < 0.05$, $\eta^2_p = 0.10$ (see Table 2). Post-hoc analyses were run to explore this interaction in depth. This interaction was due to participants performing better in the headphone condition when it was presented second rather than first ($p < .05$), and participants being better in the speaker condition when it was presented first rather than second. This significant interaction was unexpected.

Table A1

*Percentage of trials containing errors in Experiments 1 and 2. Standard deviations are displayed in parentheses.*

| | Headphones First | | Speakers First | |
|---|---|---|---|---|
| Congruency | Headphones | Speakers | Headphones | Speakers |
| Experiment 1 | | | | |
| Congruent | 0.25 (0.83) | 0.49 (1.09) | 0.24 (0.80) | 0.18 (0.74) |
| Incongruent | 0.63 (1.17) | 1.26 (2.04) | 0.87 (1.43) | 0.37 (0.91) |
| Silence | 0.19 (0.58) | 0.44 (0.96) | 0.55 (1.12) | 0.38 (1.08) |
| Experiment 2 | | | | |

| | | | | |
|---|---|---|---|---|
| Congruent | 0.94 (1.42) | 1.30 (2.03) | 1.21 (1.92) | 1.31 (1.79) |
| Incongruent | 1.42 (2.14) | 2.39 (2.90) | 1.46 (2.28) | 2.23 (3.41) |
| Tone | 1.01 (1.63) | 1.13 (1.65) | 1.07 (1.60) | 1.09 (1.60) |

**Experiment 2**

A mixed-model ANOVA was used to analyze the results. There was a significant main effect of auditory distraction, $F$ (1.83, 120.55) = 9.27, $p < 0.01$, $\eta^2_p = 0.12$, with incongruent > congruent = control. However, the main effects of location, $\eta^2_p = 0.05$, and task order, $\eta^2_p < 0.01$, were not significant, nor was the auditory distraction by task order interaction, $\eta^2_p < 0.01$, the location by task order interaction, $\eta^2_p < 0.01$, the location by auditory distraction interaction, $\eta^2_p = 0.03$, or the location by auditory distraction by task order interaction, $\eta^2_p < 0.01$ (see Table A1).

**Experiment 3**

A 3 (task order) x 3 (location) x 3 (auditory distraction) mixed-model ANOVA was used to examine error rates. There was no significant main effect of location, $\eta^2_p = 0.03$, or task order, $\eta^2_p = 0.02$; however, there was a main effect of auditory distraction, $F$ (1.78, 151.14) = 6.12, $p < .01$, $\eta^2_p = 0.07$, congruent < neutral = incongruent, and a significant location by task order interaction $F$ (4) = 4.27, $p < .01$, $\eta^2_p = 0.09$. The location by auditory distraction interaction, $\eta^2_p = 0.02$, auditory distraction by task order interaction ($\eta^2_p = 0.01$), and location by auditory distraction by task order interactions were not significant, $\eta^2_p = 0.02$. A 3 (location) x 3 (task order) repeated-measures ANOVA was run in order to examine the significant location by task order interaction. The only version of the task in which location had any impact was Version B (Middle, Back, Front), $F$ (2, 54) = 7.82, $p < .01$, $\eta^2_p = 0.22$, middle > front (see Table A2).

Table A2

Percentage of trials containing errors in Experiment 3. Standard deviations are displayed in parentheses.

| Version | Location | Congruent | Incongruent | Neutral |
|---|---|---|---|---|
| | Front | 0.63 (1.15) | 1.21 (1.68) | 0.37 (0.76) |
| Version A | Middle | 0.31 (0.72) | 0.51 (0.99) | 0.39 (0.93) |

| | | | | |
|---|---|---|---|---|
| | Back | 0.38 (0.77) | 0.70 (1.19) | 0.94 (1.30) |
| | Front | 0.34 (1.17) | 0.55 (1.02) | 0.27 (0.86) |
| Version B | Middle | 0.96 (1.24) | 1.22 (1.97) | 1.21 (1.38) |
| | Back | 0.47 (0.98) | 1.08 (1.19) | 0.80 (0.95) |
| | Front | 0.43 (0.94) | 0.65 (0.94) | 0.50 (1.29) |
| Version C | Middle | 0.37 (0.77) | 1.06 (1.66) | 0.95 (1.30) |
| | Back | 0.64 (1.27) | 0.93 (2.40) | 1.01 (1.28) |

**Experiment 4**

A 3 (auditory distraction) x 5 (location) repeated-measures ANOVA was used to analyze errors. There was a significant main effect of auditory distraction, $F$ (12, 104) = 12.59, $p < .01$, $\eta^2_p = 0.20$. There was no main effect of location, $\eta^2_p = 0.02$, nor a significant location by auditory distraction interaction, $\eta^2_p = 0.02$ (see Table A3).

Table A3

Percentage of trials containing errors in Experiment 4. Standard deviations are displayed in parentheses.

| Location | Congruent | Incongruent | Neutral |
|---|---|---|---|
| L90° | 0.24 (0.99) | 0.63 (1.57) | 0.74 (2.26) |
| L45° | 0.40 (1.24) | 1.47 (2.43) | 0.88 (2.09) |
| Center | 0.25 (1.05) | 1.04 (2.19) | 0.58 (1.74) |
| R45° | 0.25 (1.02) | 1.05 (2.05) | 0.49 (2.17) |
| R90° | 0.08 (0.57) | 1.48 (2.38) | 0.59 (1.76) |
