# Supplementary material for: Spatial Integration and the Underlying Mechanisms of Cross-Modality Interference
Source: J Cogn. 2018 Jan 10;2(1):2. doi: 10.5334/joc.5 (PMC6646940; doi:10.5334/joc.5)
Supplement: Appendix B. — Bayesian Analyses. [file joc-1-1-5-s2.pdf]

Appendix B

Bayesian Analyses

For Experiment 1, we conducted a paired samples *t*-test, $t\ (62) = -1.32$, to compare the mean difference scores in the speakers and headphones condition. The data yield a JSZ $\beta_{01} = 4.33$, indicating the observation of the data should shift one's odds in favor of the null hypothesis by a factor of approximately 4. By Jeffreys's (1961) conventions, this is considered moderate evidence for the null hypothesis.

For Experiment 2, we again conducted a paired samples *t*-test, $t\ (67) = .53$, to compare the mean difference scores in the speakers and headphones condition. The data yield a JSZ $\beta_{01} = 9.15$, indicating the observation of the data should shift one's odds in favor of the null hypothesis by a factor of approximately 9. By Jeffreys's (1961) conventions, this is considered moderate to strong evidence for the null hypothesis.

For Experiment 3, we conducted three paired samples *t*-tests to compare the mean difference scores between the Front and Back speaker locations, $t\ (87) = -.86$, the Front and Middle speaker locations, $t\ (87) = -.55$, and the Middle and Back speaker locations, $t\ (87) = -.57$. The data yield a JSZ $\beta_{01} = 8.27$, JSZ $\beta_{01} = 10.22$, and JSZ $\beta_{01} = 10.15$ respectively, indicating the observation of the data should shift one's odds in favor of the null hypothesis by a factor of approximately 8 and 10. By Jeffreys's (1961) conventions, this is considered strong evidence for the null hypothesis.

Lastly, for Experiment 4, we conducted three paired samples *t*-tests to compare the mean difference scores between the L90° and Center locations, $t\ (51) = -1.10$, the R90° and Center locations, $t\ (51) = -.43$, and the L90° and R90° locations, $t\ (51) = -1.07$. The data yield a JSZ $\beta_{01} = 5.15$, JSZ $\beta_{01} = 8.49$, and JSZ $\beta_{01} = 5.35$ respectively, indicating the observation of the data should shift one's odds in favor of the null hypothesis by a factor of approximately 5, 8, and 5. By Jeffreys's (1961) conventions, this is considered moderate evidence for the null hypothesis. We did not compare R45° and L45° as participants did not seem to be able to easily distinguish between the 45° and 90° condition. Also, we wanted to ensure that there were no differences based on which side the sound appeared to be coming from. In Experiment 3, the sound came from speakers located on either side of the participant; thus it seemed to be coming from both sides simultaneously. In Experiment 4 the sounds appear to be from either the left or the right side.
